# One-year quality of life among post-hospitalization COVID-19 patients

**DOI:** 10.3389/fpubh.2023.1236527

**Published:** 2023-10-06

**Authors:** Ignacio Pérez Catalán, Celia Roig Martí, Sergio Fabra Juana, Elena Domínguez Bajo, Germán Herrero Rodríguez, Ana Segura Fábrega, María Varea Villanueva, Sofía Folgado Escudero, María José Esteve Gimeno, Daniela Palomo de la Sota, Alejandro Cardenal Álvarez, María Lidón Mateu Campos, Jorge Usó Blasco, José Manuel Ramos Rincón

**Affiliations:** ^1^Internal Medicine Service, University General Hospital of Castellon, Castellón de la Plana, Spain; ^2^Internal Medicine Service, Hospital of Vinaroz, Vinaroz, Spain; ^3^Internal Medicine Service, Hospital of Mendaro, Mendaro, Spain; ^4^Intensive Care Unit, University General Hospital of Castellon, Castellón de la Plana, Spain; ^5^Internal Medicine Service, University General Hospital of Alicante, Alicante, Spain; ^6^Department of Clinical Medicine, Miguel Hernández University of Elche, Elche, Spain

**Keywords:** COVID-19, long COVID, post-acute COVID-19 syndrome, quality of life, SARS-COV-2

## Abstract

**Introduction:**

The long-term effects of SARS-CoV-2 are unclear, as are the factors influencing the evolution. Objective: to assess health-related quality of life 1 year after a hospital admission due to COVID-19 and to identify factors that may influence it.

**Materials and methods:**

Retrospective observational study in a tertiary hospital from March 2021 to February 2022. Inclusion criteria: ≥18 years old and admitted for SARS-CoV-2 infection. Exclusion criteria: death, not located, refusal to participate, cognitive impairment, and language barrier. Variables: demographic data, medical history, clinical and analytical outcomes during hospital admission, treatment received, and vaccination against SARS-CoV-2 following admission. Participants were interviewed by phone 1 year after admission, using the SF-36 quality of life questionnaire.

**Results:**

There were 486 included patients. The domains yielding the lowest scores were general health (median 65%, interquartile range [IQR] 45–80), vitality (median 65%, IQR 45–80), and mental health (median 73.5%, IQR 60–100). Multivariable analysis showed that female sex and fibromyalgia/fatigue had a negative influence on all domains. Obesity was associated with worse outcomes in physical functioning, physical role, bodily pain, and vitality. Other factors associated with worse scores were an older age in physical functioning and high age-adjusted Charslon comorbidity in physical functioning and general health. Age was associated with better results in emotional role and High C-reactive protein at admission on vitality.

**Conclusion:**

One year after admission for COVID-19, quality of life remains affected, especially the domains of general health, vitality, and mental health. Factors associated with worse outcomes are female sex, fibromyalgia/chronic fatigue, and obesity.

## 1. Introduction

To date, severe acute respiratory syndrome coronavirus 2 (SARS-CoV-2) has caused 676,609,955 confirmed cases and at least 6,881,955 deaths worldwide (1). The pathophysiology and clinical forms of the disease during its acute phase are already well known (2), but its long-term evolution is more uncertain, and the factors determining it, even more so. Long COVID, defined by the World Health Organization (WHO) in October 2021 as the presence of symptoms 3 months after SARS-CoV-2 infection, with a minimum duration of 2 months, which cannot be explained by an alternative diagnosis (3), now represents a significant challenge for health systems given its high prevalence, its great impact on quality of life, and the dearth of knowledge regarding its etiopathogenesis, predisposing factors, and even treatment. In addition, long COVID, also known as post-COVID condition or post-acute sequelae of COVID-19, can affect any organ system, including the central and peripheral nervous system and the cardiovascular, respiratory, or digestive systems, among others (4–7).

A recent meta-analysis in 1.2 million patients who had had a symptomatic SARS-CoV-2 infection showed that around 6.2% of them had symptoms associated with long COVID 3 months after infection (8). The mean duration of these symptoms was 9 months in those who required hospital admission and 4 months in those who did not (8). Although fatigue syndromes after infection have been previously described with other microorganisms, such as Epstein–Barr virus and cytomegalovirus, their pathogenesis is still unknown, and treatment is only symptomatic (9). However, as is the case after these infections, the long COVID syndrome may be very similar and even difficult to differentiate from myalgia encephalomyelitis/chronic fatigue syndrome (ME/CFS).

Thus, this study aims to assess health-related quality of life 1 year after a hospital admission due to SARS-CoV-2 infection and to identify factors that may influence it.

## 2. Materials and methods

### 2.1. Study design, setting, and participants

**Figure 1:**
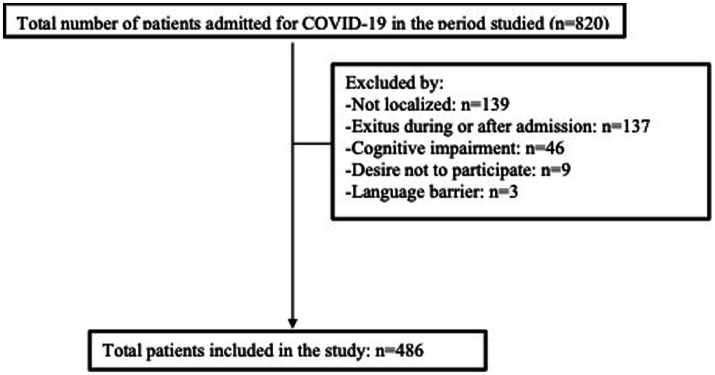
Flowchart.

This retrospective observational study was performed in the city of Castellón (Spain), in a tertiary hospital with a catchment population of 283,000 inhabitants, from March 2021 to February 2022. Eligible patients were adults (≥ 18 years) admitted to the infectious diseases unit due to SARS-CoV-2 infection from March 2020 to February 2022, confirmed by real-time polymerase chain reaction (RT-PCR) or antigen test. Exclusion criteria were: died during the first admission or during follow-up (*n* = 137), could not be located at the time of the interview (*n* = 139), refused to participate (*n* = 9), presented prior to infection notable cognitive impairment at the time of the interview (*n* = 46), or had a language barrier (*n* = 3; Figure 1).

### 2.2. Variables

Participants’ electronic medical records (EMRs) were reviewed using Orion Clinic software (Council for Universal Health Care and Public Health, Valencian Community, Spain). Data collected included *demographic variables* (age, sex), *medical history* [comorbidities including obesity, defined as body mass index ≥30 kg/m^2^, and age-adjusted Charlson comorbidity index (with higher scores indicating more comorbidity)], *clinical outcomes* [length of hospital stay, evolution to acute respiratory distress syndrome (ARDS), need for admission to the intensive care unit (ICU), type of respiratory support required, need for FiO_2_ (fraction of inspired oxygen), and Pa/FiO_2_ ratio on admission and extreme values during the hospital stay], *laboratory test results* [lymphocyte values, C-reactive protein (CRP), ferritin, and IL-6 and D-dimer at admission and extremes during the hospital stay], *treatment* (systemic corticosteroid therapy during admission and total days of corticosteroid therapy), *vaccination against SARS-CoV-2 following the hospital admission* (yes/no).

Following recruitment and provision of informed consent, the 36-item Short Form Survey (SF-36) on health-related quality of life questionnaire was administered by telephone by the investigators (all internal medicine specialists) 1 year after hospital discharge. The SF-36 evaluates eight domains, including physical functioning, physical role limitations, bodily pain, general health perceptions, energy/vitality, social functioning, emotional role limitations, and mental health (10). For each domain, a percentage value is generated, with higher scores indicating better quality of life in that domain.

Outcome variables were the score in the eight domains of the SF-36.

### 2.3. Statistical analysis

Statistical analysis was performed using SPSS software (version 23, IBM). First, a descriptive study was performed: quantitative variables were described as means (standard deviation, SD) or medians (interquartile range, IQR), depending on the normality of their distribution, and qualitative variables were described as absolute or relative frequencies. To test the association between the outcomes and the quantitative explanatory variables, the Pearson or Spearman correlation tests were performed, as appropriate. To compare the scores in each domain of the SF-36 test between the two groups of qualitative variables, the Mann–Whitney U test was used. The Bonferroni test was used to correct for multiple comparisons, so that taking into account a *p* = 0.05 and the fact that 42 variables were studied in the univariate study, only *p* < 0.0012 were considered statistically significant. Subsequently, a multivariable analysis was performed using multiple linear regression. The model included the variables that had shown a significant association with the outcome in the univariable analysis, plus sex and age.

## 3. Results

### 3.1. Study sample

A total of 486 patients were included (Figure 1). Their mean age was 61 years (SD 14), and 194 were women (39.9%). The review of the medical history showed that 111 (22.8%) were smokers or ex-smokers, 205 (44.2%) hypertensive, and 153 (31.5%) obese. The median age-adjusted Charlson comorbidity index was 2 (IQR 1–3). Median length of hospital stay was 10 days (IQR 6–15), and 100 patients (20.6%) required ICU admission, with a median stay in the unit of 6 days (IQR 4–10). ARDS was diagnosed in 193 patients (39.7%), and 93 (19.1%) required non-invasive—and 17 (3.5%) invasive—mechanical ventilation. Systemic corticosteroid therapy was administered to 432 (88.9%) patients during admission, with a median duration of 36 days (IQR 19–49). Of the total sample, 398 participants (81.9%) subsequently completed the vaccination regimen recommended at that time against SARS-CoV-2. Table 1 presents the results for FiO_2_, the Pa/FiO_2_ ratio, laboratory variables, and other descriptive indicators.

**Table 1:** Descriptive analysis.

	*n* = 486
Antecedents, *n* (%)	
*Age*, average (SD) (*n* = 486)	61 (14)
*Female*	194 (39.9)
*Smoker (and ex-smoker)*	111 (22.8)
*Hypertension*	205 (44.2)
*Dyslipemia*	140 (28.8)
*Anxiety*	56 (11.5)
*Depression*	27 (5.6)
*Fibromyalgia/chronic fatigue*	13 (2.7)
*Obesity (BMI > 30)*	153 (31.5)
*Ischemic cardiopathy*	14 (2.9)
*Cardiac insufficiency*	17 (3.5)
*COPD*	5 (1)
*Chronic bronchitis*	8 (1.6)
*Asthma*	4 (0.8)
*Chronic renal disease*	14 (2.9)
*Diabetes*	71 (14.6)
*Diabetes with target organ damage*	11 (2.3)
*Age-adjusted Charlson Comorbidity Index*, median (IQR)	2 (1–3)
Clinical evolution	
*PaO2/FiO2 at admission, median (IQR) (n* = 385*)*	333 (300–373)
*FiO2 at admission (%), median (IQR) (n = 486)*	21 (21–21)
*Minimum PaO2/FiO2, median (IQR) (n = 381)*	300 (145–357)
*Maximum FiO2 (%), median (IQR) (n = 486)*	*32 (21–60)*
*ARDS, n (%)*	193 (39.7)
*Intensive care unit, n (%)*	100 (20.6)
*CPAP-Helmet, n (%)*	93 (19.1)
*High flow oxygen, n (%)*	26 (5.3)
*Mechanical ventilation, n (%)*	17 (3.5)
*Hospital stay (days), median (IQR)*	10 (6–15)
*Stay in the Intensive Care Unit (days), median (IQR)*	6 (4–10)
Analytical parameters, median (IQR)	
*Lymphopenia at admission (/μL) (n = 484)*	990 (712–1320)
*RCP at admission (mg/L) (n = 486)*	64 (30–116)
*Ferritin at admission (mcg/L) (n = 443)*	482 (258–886)
*IL-6 at admission (ng/L) (n = 285)*	34 (16–60)
*d-dimer at admission (ng/mL) (n = 431)*	610 (380–1080)
*Minimum lymphocytes during admission (/μL) (n = 484)*	720 (520–1097)
*Maximum RCP during admission (mg/L) (n = 485)*	83 (40–136)
*Maximum ferritin during admission (mcg/L) (n = 447)*	655 (354–1189)
*Maximum IL-6 during admission (ng/L) (n = 357)*	38 (16–67)
*Maximum d-dimer during admission (ng/mL) (n = 480)*	940 (570–2120)
Treatment	
Systemic corticosteroids during admission, *n (%)*	432 (88.9)
Total days of corticotherapy, median (IQR; *n* = 486)	36 (19–49)
SARS-CoV-2 vaccination after admission, *n* (%)	398 (81.9)

### 3.2. SF-36 quality of life scores

According to each domain of the SF-36, median scores were as follows: physical functioning, 95% (IQR 70–100); physical role limitations, 100% (IQR 75–100); bodily pain, 90% (IQR 66.9–100); general health, 65% (IQR 45–80); vitality, 65% (IQR 45–80); social functioning, 100% (IQR 87.5–100); emotional role limitations, 100% (IQR 100–100); and mental health, 73.5% (IQR 60–100).

### 3.3. Association between explanatory variables and SF-36 quality of life scores

The influence of each of the variables studied on the results of each of the eight domains of the SF-36 test was analyzed. In the univariable study, female sex, obesity, and a history of fibromyalgia/chronic fatigue were significantly associated with poorer quality of life in all domains of the SF-36. A history of anxiety and depression also showed a negative influence in most domains. In contrast, the greater inflammatory response, represented especially by high levels of ferritin at and during admission, was significantly associated with better scores in some domains. Systemic treatment with corticosteroids during admission showed some protective effect in terms of body pain, regardless of the duration of treatment, although after correction by the Bonferroni test it did not show statistical significance and also showed no relationship with the rest of the domains. The rest of the results are presented in Tables 2, 3.

**Table 2:** Association between qualitative variables and median scores for each SF-36 domain 1 year after hospital admission for COVID-19.

Qualitative variables	Comparison of median scores and IQR (in brackets) in each SF-36 domain, according to dichotomous explanatory variables (no/yes; Mann–Whitney U test)																							
	Physical functioning			Physical role			Bodily pain			General health			Vitality			Social functioning			Emotional role			Mental health		
	*No*	*Yes*	*p*	*No*	*Yes*	*p*	*No*	*Yes*	*p*	*No*	*Yes*	*p*	*No*	*Yes*	*p*	*No*	*Yes*	*p*	*No*	*Yes*	*p*	*No*	*Yes*	*p*
Medical history																								
Female	95 (80–100)	80 (49–95)	**<0.001**	100 (100–100)	100 (0–100)	**<0.001**	100 (80–100)	70 (45–100)	**<0.001**	70 (60–80)	55 (35–70)	**<0.001**	70 (55–85)	55 (35–75)	**<0.001**	100 (88–100)	100 (62–100)	**<0.001**	100 (100–100)	100 (67–100)	**<0.001**	80 (64–88)	64 (48–80)	**<0.001**
Smoker/ex-smoker	90 (70–100)	95 (70–100)	0.600	100 (75–100)	100 (75–100)	0.700	90 (60–100)	90 (67–100)	0.910	65 (45–80)	65 (45–75)	0.810	65 (45–80)	65 (45–80)	0.960	100 (75–100)	100 (87–100)	0.880	100 (100–100)	100 (100–100)	0.071	72 (60–88)	76 (60–84)	0.670
Hypertension	95 (75–100)	90 (57–100)	**<0.001**	100 (75–100)	100 (75–100)	0.870	100 (67–100)	90 (57–100)	0.610	70 (45–80)	65 (45–75)	0.082	70 (45–80)	65 (45–80)	0.710	100 (87–100)	100 (87–100)	0.540	100 (100–100)	100 (100–100)	0.860	72 (60–84)	76 (60–88)	0.150
Dyslipidemia	95 (70–100)	90 (55–100)	0.044	100 (78–100)	100 (75–100)	0.300	90 (67–100)	90 (57–100)	0.980	65 (45–80)	65 (45–75)	0.180	65 (45–80)	67 (45–80)	0.950	100 (87–100)	100 (75–100)	0.790	100 (100–100)	100 (100–100)	0.920	72 (59–84)	76 (60–88)	0.270
Anxiety	95 (70–100)	77 (55–95)	**0.001**	100 (75–100)	100 (0–100)	0.110	100 (67–100)	80 (58–100)	0.130	70 (45–80)	60 (36–70)	0.006	70 (45–84)	50 (35–65)	**<0.001**	100 (87–100)	88 (63–100)	0.015	100 (100–100)	100 (33–100)	0.003	76 (60–88)	64 (53–79)	**<0.001**
Depression	95 (70–100)	70 (55–90)	0.005	100 (75–100)	100 (75–100)	0.720	90 (67–100)	90 (60–100)	0.700	65 (45–80)	55 (30–75)	0.006	70 (45–80)	45 (30–65)	**0.001**	100 (87–100)	100 (38–100)	0.270	100 (100–100)	100 (67–100)	0.400	76 (60–88)	68 (56–84)	0.250
Fibromyalgia/chronic fatigue	95 (70–100)	40 (22–55)	**<0.001**	100 (77–100)	0 (0–100)	**<0.001**	100 (67–100)	45 (23–62)	**<0.001**	65 (45–80)	30 (17–42)	**<0.001**	70 (45–80)	30 (17–37)	**<0.001**	100 (87–100)	63 (37–87)	**<0.001**	100 (100–100)	33 (0–100)	**0.001**	76 (60–88)	48 (42–66)	**<0.001**
Obesity (BMI > 30 kg/m^2^)	95 (80–100)	80 (50–95)	**<0.001**	100 (100–100)	100 (0–100)	**<0.001**	100 (70–100)	80 (52–100)	**<0.001**	70 (50–80)	60 (40–75)	**0.001**	70 (50–85)	60 (40–75)	**<0.001**	100 (87–100)	100 (75–100)	0.010	100 (100–100)	100 (67–100)	0.015	76 (60–88)	72 (54–84)	**0.027**
Ischemic cardiopathy	95 (70–100)	75 (54–95)	0.040	100 (75–100)	100 (62–100)	0.560	95 (61–100)	80 (68–100)	0.340	65 (45–80)	47 (40–64)	0.037	65 (45–80)	70 (54–85)	0.450	100 (87–100)	100 (84–100)	0.570	100 (100–100)	100 (100–100)	0.600	72 (60–87)	82 (67–92)	0.140
Cardiac insufficiency	95 (70–100)	75 (10–95)	0.004	100 (75–100)	100 (87–100)	0.820	100 (67–100)	70 (54–100)	0.130	65 (45–80)	55 (40–65)	0.015	65 (45–80)	70 (57–82)	0.310	100 (87–100)	100 (81–100)	0.870	100 (100–100)	100 (100–100)	0.400	72 (58–84)	80 (66–92)	0.120
COPD	95 (70–100)	90 (57–95)	0.410	100 (75–100)	100 (87–100)	0.590	90 (67–100)	70 (21–90)	0.130	65 (45–80)	75 (37–82)	0.770	65 (45–80)	85 (47–95)	0.170	100 (87–100)	100 (81–100)	0.480	100 (100–100)	100 (100–100)	0.270	72 (60–88)	80 (56–84)	0.940
Chronic bronchitis	95 (70–100)	80 (59–99)	0.420	100 (75–100)	100 (25–100)	0.950	90 (67–100)	90 (34–100)	0.820	65 (45–80)	60 (46–74)	0.520	65 (45–80)	75 (52–84)	0.330	100 (87–100)	100 (81–100)	0.550	100 (100–100)	100 (100–100)	0.540	72 (60–88)	76 (61–83)	0.930
Asthma	95 (70–100)	47 (21–77)	0.023	100 (75–100)	0 (0–75)	0.012	90 (67–100)	34 (22–86)	0.064	65 (45–80)	25 (20–56)	0.020	65 (45–80)	20 (15–44)	0.008	100 (87–100)	63 (41–94)	0.060	100 (100–100)	50 (0–100)	0.084	75 (60–88)	54 (37–74)	0.110
Chronic kidney disease	95 (70–100)	70 (12–91)	0.006	100 (75–100)	100 (0–100)	0.280	90 (67–100)	70 (39–100)	0.270	65 (45–80)	47 (32–66)	0.040	65 (45–80)	55 (30–76)	0.240	100 (87–100)	100 (47–100)	0.400	100 (100–100)	100 (67–100)	0.420	73 (60–88)	70 (39–85)	0.320
Diabetes	95 (70–100)	90 (60–100)	0.240	100 (75–100)	100 (0–100)	0.450	90 (67–100)	100 (55–100)	0.750	65 (45–80)	65 (45–80)	0.600	65 (45–80)	65 (40–85)	0.580	100 (87–100)	100 (75–100)	0.930	100 (100–100)	100 (100–100)	0.580	75 (60–84)	72 (52–88)	0.870
Diabetes with target organ damage	95 (70–100)	75 (35–95)	0.044	100 (75–100)	100 (75–100)	0.560	100 (67–100)	68 (57–90)	0.110	65 (45–80)	45 (35–60)	0.039	65 (45–80)	65 (45–85)	0.890	100 (87–100)	100 (87–100)	0.690	100 (100–100)	100 (100–100)	0.418	72 (60–84)	84 (68–92)	0.140
Clinical outcomes																								
ARDS	95 (70–100)	90 (70–100)	0.830	100 (75–100)	100 (100–100)	0.310	90 (67–100)	100 (62–100)	0.620	65 (45–75)	65 (50–80)	0.780	65 (40–80)	70 (50–85)	0.054	100 (75–100)	100 (87–100)	0.580	100 (100–100)	100 (100–100)	0.350	72 (56–84)	76 (62–88)	0.091
ICU admission	95 (70–100)	95 (66–100)	0.950	100 (75–100)	100 (75–100)	0.710	95 (67–100)	90 (57–100)	0.500	65 (45–80)	70 (50–80)	0.230	65 (45–80)	70 (46–89)	0.041	100 (87–100)	100 (75–100)	0.370	100 (100–100)	100 (67–100)	0.054	74 (60–85)	73 (60–88)	0.540
Helmet-CPAP	95 (70–100)	95 (65–100)	0.990	100 (75–100)	100 (75–100)	0.620	100 (67–100)	90 (57–100)	0.370	65 (45–80)	70 (47–80)	0.330	65 (45–80)	70 (45–85)	0.086	100 (87–100)	100 (75–100)	0.470	100 (100–100)	100 (67–100)	0.041	76 (60–86)	72 (58–88)	0.640
High-flow oxygen	95 (66–100)	95 (81–100)	0.300	100 (75–100)	100 (100–100)	0.170	90 (61–100)	100 (77–100)	0.260	65 (45–80)	70 (60–80)	0.161	65 (45–80)	70 (60–86)	0.035	100 (87–100)	100 (87–100)	0.980	100 (100–100)	100 (100–100)	0.360	72 (57–84)	80 (63–88)	0.210
Mechanical ventilation	95 (70–100)	90 (60–100)	0.730	100 (75–100)	100 (50–100)	0.950	90 (67–100)	80 (46–100)	0.250	65 (45–80)	70 (52–87)	0.500	65 (45–80)	75 (39–90)	0.370	100 (87–100)	87 (69–100)	0.140	100 (100–100)	100 (100–100)	0.880	72 (60–88)	80 (56–88)	0.720
Systemic corticosteroids during admission	90 (45–100)	95 (70–100)	0.170	100 (25–100)	100 (75–100)	0.210	80 (57–100)	100 (67–100)	0.023	62 (35–76)	65 (45–80)	0.330	60 (39–85)	70 (45–80)	0.340	100 (75–100)	100 (87–100)	0.061	100 (100–100)	100 (100–100)	0.820	76 (51–88)	72 (60–84)	0.980
SARS-CoV-2 vaccination after admission	95 (65–100)	95 (71–100)	0.960	100 (75–100)	100 (76–100)	0.670	90 (60–100)	90 (68–100)	0.200	70 (45–80)	65 (45–75)	0.410	70 (50–85)	70 (50–80)	0.360	100 (75–100)	100 (88–100)	0.450	100 (100–100)	100 (100–100)	0.820	80 (60–88)	75 (60–84)	0.180

ARDS, Acute respiratory distress syndrome; BMI, Body mass index; COPD, Chronic obstructive pulmonary disease; ICU, Intensive care unit; and SF-36, 36-item Short Form health survey.

Values in bold have reached statistical significance.

**Table 3:** Association between quantitative variables and quality of life outcomes, according to the different domains of the SF-36 test 1 year after hospital admission.

Quantitative variables	Correlation ^*^ between quantitative variables and quality of life outcomes, according to SF-36 domain															
	Physical functioning		Physical role		Bodily pain		General health		Vitality		Social functioning		Emotional role		Mental health	
	*r_s_*	*p*	*r_s_*	*p*	*r_s_*	*p*	*r_s_*	*p*	*r_s_*	*p*	*r_s_*	*p*	*r_s_*	*p*	*r_s_*	*p*
Medical history																
Age^*^ (*n* = 486)	−0.302	**<0.001**	−0.036	0.440	−0.054	0.230	−0.132	0.004	−0.032	0.480	0.025	0.590	0.086	0.059	0.052	0.250
Age-adjusted Charlson Comorbidity Index (*n* = 486)	−0.294	**<0.001**	−0.059	0.200	−0.077	0.092	−0.166	**<0.001**	−0.043	0.350	0.006	0.900	0.067	0.140	0.038	0.410
Clinical outcomes																
Hospital stay (days) (*n* = 486)	−0.188	**<0.001**	−0.650	0.150	−0.091	0.045	−0.074	0.100	−0.016	0.720	−0.084	0.064	−0.050	0.27	0.038	0.410
ICU admission (days) (*n* = 100)	−0.043	0.670	0.067	0.510	−0.017	0.870	0.016	0.870	0.011	0.920	−0.047	0.640	0.043	0.670	0.029	0.780
PaO_2_/FiO_2_ at admission (*n* = 385)	0.124	0.015	0.005	0.920	0.047	0.360	0.069	0.180	−0.035	0.490	0.038	0.460	0.040	0.430	−0.040	0.430
FiO_2_ at admission (%) (*n* = 486)	−0.098	0.031	−0.021	0.640	−0.084	0.064	−0.024	0.590	0.058	0.200	−0.019	0.670	−0.034	0.460	−0.022	0.620
Min PaO_2_/FiO_2_ (*n* = 381)	0.017	0.750	−0.069	0.180	−0.020	0.700	−0.033	0.520	−0.132	0.010	−0.008	0.880	0.023	0.660	−0.116	0.023
Max FiO_2_ (%) (*n* = 486)	−0.145	**0.001**	−0.006	0.900	−0.029	0.520	−0.054	0.230	0.043	0.350	−0.018	0.690	−0.048	0.290	0.035	0.440
Analytical parameters																
Lymphopenia at admission (/μL) (*n* = 484)	−0.021	0.640	−0.084	0.064	−0.077	0.089	−0.060	0.190	−0.096	0.034	−0.031	0.500	−0.070	0.880	−0.062	0.180
CRP at admission (mg/L) (*n* = 486)	0.017	0.720	0.089	0.049	0.075	0.100	0.086	0.058	0.150	**0.001**	0.033	0.470	0.057	0.210	0.114	0.010
Ferritin at admission (μg/L) (*n* = 443)	0.233	**<0.001**	0.127	0.007	0.185	**<0.001**	0.170	**<0.001**	0.222	**<0.001**	0.164	**0.001**	0.135	0.005	0.211	**<0.001**
IL-6 at admission (ng/L) (*n* = 285)	0.043	0.470	0.114	0.055	0.088	0.140	0.054	0.360	0.138	0.020	−0.017	0.780	0.066	0.270	0.106	0.073
D-dimer at admission (ng/mL) (*n* = 431)	−0.068	0.160	−0.031	0.520	−0.034	0.480	0.003	0.950	0.030	0.540	−0.014	0.770	0.009	0.850	0.008	0.880
Min lymphocytes during admission (/μL) (*n* = 484)	0.045	0.320	−0.036	0.430	−0.039	0.390	−0.051	0.270	−0.069	0.130	−0.006	0.890	0.003	0.940	−0.094	0.039
Max RCP during admission (mg/L) (*n* = 485)	−0.001	0.980	0.072	0.110	0.088	0.054	0.070	0.120	0.135	0.003	0.023	0.610	0.045	0.330	0.112	0.014
Max ferritin during admission (mcg/L) (*n* = 447)	0.169	**<0.001**	0.100	0.028	0.167	**<0.001**	0.153	**0.001**	0.177	**<0.001**	0.171	**<0.001**	0.103	0.025	0.188	**<0.001**
Max IL-6 during admission (ng/L) (*n* = 357)	−0.035	0.510	0.001	0.980	0.041	0.440	−0.028	0.600	0.088	0.098	−0.063	0.240	0.004	0.950	0.031	0.570
Max d-dimer during admission (ng/mL) (*n* = 480)	−0.124	0.006	−0.058	0.200	−0.034	0.460	<0.001	1.000	0.026	0.580	−0.046	0.320	−0.032	0.490	0.028	0.540
Total days of corticosteroid treatment (*n* = 486)	−0.049	0.280	−0.043	0.350	0.060	0.190	−0.031	0.490	0.001	0.980	−0.014	0.750	−0.028	0.530	0.009	0.840

CRP, C reactive protein; IQR, Interquartile range. ^*^Correlation presented as Spearman’s rho (rs), except in the case of age, where it is Pearson’s correlation coefficient.

Values in bold have reached statistical significance.

The multivariable model included all variables showing a statistically significant association in the univariable study and was adjusted for sex and age (Table 4). Both female sex and history of fibromyalgia/chronic fatigue continued to show a significant and negative association with all domains of the SF-36 test. Obesity had a smaller influence and was related to worse outcomes in physical functioning (*p* = 0.002), physical role (*p* < 0.001), bodily pain (*p* = 0.040) and vitality (*p* = 0.009). Other factors associated with worse scores on a particular domain of the SF-36 were: an older age in physical functioning (*p* = 0.047) and high age-adjusted Charslon comorbidity index in physical functioning (*p* = 0.013) and general health (*p* = 0.027). In contrast, older age was associated with better results in emotional role (*p* = 0.041) and a higher RCP value at admission showed better results in vitality (*p* = 0.031). No other statistically significant associations were observed.

**Table 4:** Results of the multivariable linear regression analysis of the association between explanatory variables and quality of life domains on the SF-36.

	Physical functioning		Physical role		Bodily pain		General health		Vitality		Social functioning	Emotional role			Mental health	
Variables	β (95% CI)	*p*	β (95% CI)	*p*	β (95% CI)	*p*	β (95% CI)	*p*	β (95% CI)	*p*	β (95% CI)	*p*	β (95% CI)	*p*	β (95% CI)	*p*
Female	**−15.074 (−20.097, −10.050)**	**<0.001**	**−16.466 (−23.286, −9.645)**	**<0.001**	**−15.322 (−20.672, −9.971)**	**<0.001**	**−12.546 (−16.795, −8.298)**	**<0.001**	**−10.264 (−15.106, −5.422)**	**<0.001**	**−12.334 (−16.938, −7.731)**	**<0.001**	**−12.447 (−18.591, −6.303)**	**<0.001**	**−9.522 (−13.515, −5.529)**	**<0.001**
Hypertension	1.865 (−3.399, 7.129)	0.487	—	—	—	—	—	—	—	—	—	—	—	—	—	—
Anxiety	−2.478 (−9.327, 4.371)	0.477	—	—	—	—	—	—	−7.269 (−14.616, 0.077)	0.052	—	—	—	—	−3.250 (−8.716, 2.215)	0.243
Depression	—	—	—	—	—	—	—	—	−3.589 (−13.639, 6.461)	0.483	—	—	—	—	—	—
Fibromyalgia/chronic fatigue	**−25.666 (−39.481, −11.851)**	**<0.001**	**−28.310 (−49.099, −7.520)**	**0.008**	**−26.975 (−41.962, −11.988)**	**<0.001**	**−23.478 (−35.369, −15.586)**	**<0.001**	**−23.370 (−36.646, −10.094)**	**0.001**	**−16.190 (−29.037, −3.343)**	**0.014**	**−26.531 (−45.175, −7.887)**	**0.005**	**−12.143 (−23.173, −1.113)**	**0.031**
Obesity (BMI > 30 kg/m^2^)	**−8.192 (−13.232, −3.152)**	**0.002**	**−15.430 (−22.521, −8.338)**	**<0.001**	**−5.467 (−10.672, −0.263)**	**0.040**	−2.902 (−7.109, 1.306)	0.176	**−6.075 (−10.650, −1.500)**	**0.009**	—	—	—	—	−2.278 (−6.087, 1.532)	0.241
Age	**−0.281 (−0.557, −0.004)**	**0.047**	−0.056 (−0.289, 0.177)	0.638	−0.069 (−0.238, 0.100)	0.424	0.039 (−0.196, 0.274)	0.744	−0.088 (−0.239, 0.063)	0.254	0.064 (−0.082, 0.209)	0.390	**0.219 (0.009, 0.429)**	**0.041**	0.084 (−0.040, 0.208)	0.185
Age-adjusted Charlson Comorbidity Index	**−2.611 (−4.675, −0.547)**	**0.013**	—	—	—	—	**−1.996 (−3.768, −0.223)**	**0.027**	—	—	—	—	—	—	—	—
Length of hospital stay	−0.239 (−0.623, 0.146)	0.223	—	—	—	—	—	—	—	—	—	—	—	—	—	—
Max FiO_2_	−0.065 (−0.209, 0.079)	0.376	—	—	—	—	—	—	—	—	—	—	—	—	—	—
CRP on admission	—	—	—	—	—	—	—	**—**	**0.033 (0.003, 0.063)**	**0.031**	—	—	—	—	—	—
Ferritin at admission	0.001 (−0.005, 0.006)	0.779	—	—	0.001 (−0.006, 0.006)	0.998	0.002 (−0.003, 0.007)	0.365	0.003 (−0.002, 0.008)	0.276	0.002 (−0.003, 0.007)	0.393	—	—	0.001 (−0.003, 0.005)	0.654
Max ferritin during admission	0.000 (−0.005, 0.005)	0.961	—	—	−0.001 (−0.006, 0.004)	0.756	−0.003 (−0.007, 0.001)	0.173	−0.003 (−0.007, 0.002)	0.200	−0.003 (−0.007, 0.002)	0.204	—	—	0.000 (−0.004, 0.003)	0.825
Model parameters																
*R* ^2^	0.271		0.111		0.133		0.157		0.155		0.089		0.059		0.101	
*F* (*p*)	14.457 (<0.001)		15.090 (<0.001)		11.046 (<0.001)		11.468 (<0.001)		8.740 (<0.001)		8.492 (<0.001)		11.065 (<0.001)		6.967 (<0.001)	
Df	11, 428		4, 481		6, 433		6, 433		9, 430		5,434		3, 482		7, 432	
1-β	1		1		1		1		1		1		1		1	

BMI, Body mass index; CI, Confidence interval; CRP, C-reactive protein; ICU, Intensive care unit; SF-36, 36-item Short Form health survey; Df, Degree of freedom. “—”: not included in the multivariate study.

Values in bold have reached statistical significance.

## 4. Discussion

Our cohort of patients is made up of adults in their 60s, mainly men, without particularly high comorbidity. None of them were vaccinated against SARS-CoV-2 at the time of their admission; slightly less than half presented ARDS, and practically all of them were treated with corticosteroids. The worst quality of life outcomes were obtained in the domains of general health, vitality, and mental state, with similar results to those observed by Koullias et al. (11), who administered a simpler version of the SF-36 at 6 months after admission for coronavirus disease 2019 (COVID-19). Our results are also consistent with theirs in terms of the acceptable scores obtained in the domains referring to physical issues. Those authors also observed significantly worse results in patients who had required hospital admission compared to those who had not and to the control group. The analysis of an Italian cohort also found, on this occasion using the EQ-5D-5L quality of life survey by phone call, that at 2 years after the index admission for COVID-19, the score was worse in the mental health domain, but scores were good in the other domains, including those related to physical aspects (12). Another study in our country, Spain, used the SF-36 to assess telematically quality of life in patients admitted to the hospital for COVID-19 during the first wave (as we did), at 3 and 12 months after the onset of infection (13). They compared the results with the reference population values in Spain in 1998, observing a statistically significant decrease in the score in all domains at 3 months (especially for physical role and emotional role), and in all domains except mental health at 12 months (14). Muñoz-Corona et al. (15) also described a much more evident deterioration in the domain of physical role in patients who required hospital admission, although in this case results were probably influenced by the fact that the SF-36 test was carried out 90 days after discharge, much sooner than in the other studies mentioned, including ours.

There was evidence, based on our results and the data already published in this regard, that COVID-19, and in our case hospital admission for this disease, produces a long-term deterioration in quality of life. Moreover, understanding the predisposing factors of this deterioration is very important, since it could enable preventive interventions and help identify the most susceptible groups of patients for more intense medical follow-up. In this sense, we observed that quality of life in practically all domains, is especially compromised for a very specific patient profile: female and with a history of fibromyalgia/chronic fatigue and to a lesser extent obesity. In contrast, the severity of the disease (represented by the degree of respiratory failure, the FiO_2_ required, the type of respiratory support, and the need for ICU admission) did not appear to have an impact on subsequent quality of life. In addition, in the univariable analysis, a greater inflammatory response showed a protective effect on quality of life 1 year after hospital admission, especially elevated ferritin levels on admission and the maximum levels during the hospital stay. However, this effect did not reach statistical significance in multivariable analysis. After an extensive literature review, we found no data on how elevation of acute phase reactants during acute infection influences long-term clinical course. However, it is likely that potential contributors to Long COVID include multiple organ injury due to excessive inflammation or clotting/coagulation issues in the acute phase (16). In addition, Qu et al. (17) observed that the C-reactive protein value after hospital discharge was not associated with changes in long-term physical or mental status. These results raise the hypothesis that the long COVID would be more influenced by a certain patient profile than by the severity of the acute infection.

Different studies have tried to identify what factors influence long-term quality of life outcomes in COVID-19. Female sex is the most frequently described determinant, in keeping with our findings (11, 12, 17–22). Likewise, obesity has been described as another relevant factor (21). Other long-term determinants mentioned in the literature are advanced age, chronic diseases like diabetes, heart failure, and chronic kidney disease, hospital stay, and the need for ICU admission (17, 20–22). In our sample, only age and age-adjusted Charlson comorbidity index were also associated with worse outcomes, although in the multivariate analysis both only maintained their negative effect on physical functioning and the age-adjusted Charslon comorbidity index also in general health.

Strengths of this study include its analysis of the impact of psychological and psychiatric comorbidities, not just physical ones, on long-term quality of life after admission for COVID-19. We also report laboratory results during the acute phase of infection. We also analyzed the use of corticosteroids, since there are data that suggest a protective effect on the persistence of symptoms after infection, probably due to its anti-inflammatory effect with consequent reduction of organ and tissue damage (23). In practically all of the studies cited, these variables are not analyzed, so our data are of special interest.

On the other hand, the study also presents several limitations, such as its retrospective nature or lack of estimation of size calculation/power calculation. The absence of a control group is also a limitation, as well as the lack of reference or expected values of the SF-36 test for a population similar to ours. In addition, we also do not have the score on the SF-36 test prior to infection. Finally, as included patients were infected in the early stages of the pandemic, the protective effect that vaccination against SARS-Cov-2 could have had prior to infection could not be assessed, although a recent systematic review and meta-analysis provides strong support in that line (24). The same occurs with antiviral drugs against SARS-CoV-2, as these were not contemplated in our center’s therapeutic protocol during the period when participants were admitted. At that time, the therapeutic protocol for COVID-19 pneumonia in our hospital only contemplated systemic corticotherapy, thromboprophylaxis with low molecular weight heparins and the consideration of empirical antibiotherapy if there was suspicion of bacterial coinfection. Recent data indicate that the use of nirmatrelvir/ritonavir in acute infection would significantly decrease the subsequent incidence of long COVID (25).

## 5. Conclusion

Patients who required admission for COVID-19 in 2020 and early 2021 continued to show a diminished quality of life 1 year after hospital discharge, especially in the domains of general health, vitality, and mental health. The main factors that may influence this would be female sex, a history of fibromyalgia/chronic fatigue, and, to a lesser extent, obesity. More data are needed to evaluate the role of the inflammatory response and specifically serum ferritin in it.

## Data availability statement

The original contributions presented in the study are included in the article/supplementary material, further inquiries can be directed to the corresponding authors.

## Ethics statement

The studies involving humans were approved by Ethics and Drug Research Committee of the Castellón General University Hospital. The studies were conducted in accordance with the local legislation and institutional requirements. Written informed consent for participation was not required from the participants or the participants’ legal guardians/next of kin because informed consent was given verbally.

## Author contributions

IP and CR: conception and design of the study, writing of the manuscript, bibliographic search, data collection, and analysis and interpretation of data. SeF, ED, GH, AS, MV, SoF, ME, DP, and AC: data collection and bibliographic search. MM, JU, and JR: conception and design of the study and writing of the manuscript. All authors contributed to the article and approved the submitted version.

## Abbreviations

IQR: Interquartile range
WHO: World Health Organization
ME/CFS: Encephalomyelitis/chronic fatigue syndrome
RT-PCR: Real-time polymerase chain reaction
EMRs: Electronic medical records
ARDS: Acute respiratory distress syndrome
ICU: Intensive unit care
RCP: C-Reactive protein
SF-36: 36-Item Short Form Survey
SD: Standard deviation
BMI: Body mass index
COPD: Chronic obstructive pulmonary disease
CI: Confidence interval
COVID-19: Coronavirus disease 2019
